# A metastable structure for the compact 30‐nm chromatin fibre

**DOI:** 10.1002/1873-3468.12128

**Published:** 2016-03-30

**Authors:** Chenyi Wu, John E. McGeehan, Andrew Travers

**Affiliations:** ^1^ Molecular Biophysics LaboratoriesSchool of Biological SciencesUniversity of PortsmouthUK; ^2^ MRC Laboratory of Molecular BiologyCambridgeUK; ^3^ Department of BiochemistryUniversity of CambridgeUK

**Keywords:** 30‐nm fibre, chromatin structure, DNA supercoiling, interdigitation, nucleosome

## Abstract

The structure of compact 30‐nm chromatin fibres is still debated. We present here a novel unified model that reconciles all experimental observations into a single framework. We propose that compact fibres are formed by the interdigitation of the two nucleosome stacks in a 2‐start crossed‐linker structure to form a single stack. This process requires that the dyad orientation of successive nucleosomes relative to the helical axis alternates. The model predicts that, as observed experimentally, the fibre‐packing density should increase in a stepwise manner with increasing linker length. This model structure can also incorporate linker DNA of varying lengths.

## Abbreviations

**DNA**, deoxyribonucleic acid

**EM**, electron microscopy

**NRL**, nucleosome repeat length

The 30‐nm fibre was proposed in 1976 as the unit of the higher order packing of eukaryotic chromatin, containing the linker histone as an essential component 1. Yet, the structure of the fibre remains controversial. In particular, two apparently incompatible models, the solenoid 1 and the crossed‐linker model 2, have dominated the discussion.

In the solenoid model 1 consecutive nucleosomes (*i*,* i* + 1, *i* + 2…) of a nucleosomal array form a single helix while the linker DNAs are bent in the interior of the fibre. In contrast the crossed‐linker model 2 features two separate nucleosomal stacks with linker DNAs crossing the fibre core. Each helical nucleosomal stack thus consists of alternating nucleosomes (*i*,* i* + 2, *i* + 4…) in the array. The two models correspond, respectively, to 1‐start and 2‐start fibres.

There is now compelling structural evidence supporting the existence of 2‐start fibres. In addition to early EM images 3 a 2‐start structure is supported by Fourier analysis of images of chromatin fibres 2, chemical cross‐linking 4, the crystal structure of a tetranucleosome 5, cryo‐EM of native fibres 6, cryo‐EM structures of reconstituted fibres containing up to 24 nucleosomes 7 and cryo‐tomographic analysis of native chromatin 8, 9. Nevertheless, early studies on various native chromatin samples by photochemical dichroism 10 and X‐ray diffraction 11, 12, revealed, respectively, a small tilt of the nucleosomal disc relative to the fibre axis and narrow diffraction arcs at 110 Å. Together these observations suggested a helical structure with a low pitch, more consistent with the 1‐start model.

More recently EM and cryo‐EM measurements on the dimensions of more compact reconstituted fibres covering a wide range of linker lengths led to the proposal of a 1‐start interdigitated structure 13. Unexpectedly the data revealed a step‐wise increase in fibre‐packing density and diameter. Modelling of these fibres predicted the existence of multistart fibres with several nucleosome stacks 14, 15, 16, 17 (Table 1) but did not provide a uniform explanation for the observed step‐wise changes in fibre parameters.

**Table 1:** Dependence on linker length of calculated structural parameters for a compact 1‐start fibre. The calculated values correspond to the values for helix gyre separation, nucleosomal tilt and helix pitch angle given in the text for the same state of compaction. Linker proximity is the distance between adjacent duplex helical axes. Note that for some linker lengths this distance is less than the diameter of a duplex (2 nm) but is greater than the minimum value attained on duplex penetration (~ 1.2 nm) 19

NRL (bp)	Diameter (nm)	Nuc/11 nm	Nuc/turn	Linker proximity^a^ (nm)
177	28.3	6.5	6.5	2.87
187	30.8	7.7	7.7	2.88
197	34.9	10.5	10.5	2.62
207	37.9	11.2	11.2	2.35
217	42.2	14.6	14.6	1.71
227	45.5	15.7	15.7	1.40
237	48.9	15.8	15.8	1.40

a Average distance between immediate neighbours (linker *i* and linker *i* + 2).

NRL, nucleosome repeat length.

We present here a novel model for the condensation of the chromatin fibre that in principle resolves the current dichotomy, particularly for the most compact forms of the fibre. For these forms we propose a radical structure that has a single stack of nucleosomes and is readily interconvertible with the canonical crossed‐linker structure. Crucially it has novel features facilitating the optimal interfacial interactions between adjacent nucleosomes. We argue that the most compact form of this structure is likely metastable.

## Model building

Our starting point was to seek structural solutions for maximum fibre compaction (the amount of DNA per unit fibre volume) including the globular domain of linker histone H5, with the principal assumption that the linker DNA configuration be consistent with the crossed‐linker model. By formulating Excel Solver the procedure used published parameters from determined structures while allowing geometric variables to float within attainable limits.

The distance between two adjacent nucleosomes was set ≥ 57.6 Å centre to centre 5. Linker DNAs were modelled as straight rods of 3.04 Å per bp and 20 Å in diameter, with an allowance of ± 2 bp per linker. For the longest linkers, corresponding to 217–237‐bp nucleosome repeat lengths (NRLs), the rod was segmented in varying trajectories for approximation of smooth bending. Packing was permitted to ≥ 12 Å between helical axes of neighbouring DNA duplexes in crossovers 18, 19. We assumed that the H5 linker histone stabilises a left‐handed crossing of linker DNAs 20, 21.

Solver outputs a list of Cartesian points on a space‐helix, specifying the centres of adjacent nucleosomes. At each point an atomic model of the chromatosome (PDB ID: 4QLC) 22 is aligned with the nucleosome superhelical axis tangent to the curve and its dyad axis pointing towards the opposing nucleosomes it connects. A small adjustment to entry/exit points of the chromatosome (Fig. S2) was formulated in Solver for attaining a more optimal linker trajectory, consistent with a left‐handed crossover of entering and exiting linker DNAs. Spatial manipulations are performed using transformation matrices imbedded in the algorithm.

## Interconvertibility between related structures

We derived three archetypal structures including the canonical crossed‐linker model and two other variants, which are in principle interconvertible. In all cases the nucleosome stacks form a left‐handed coil.

**Figure 1:**
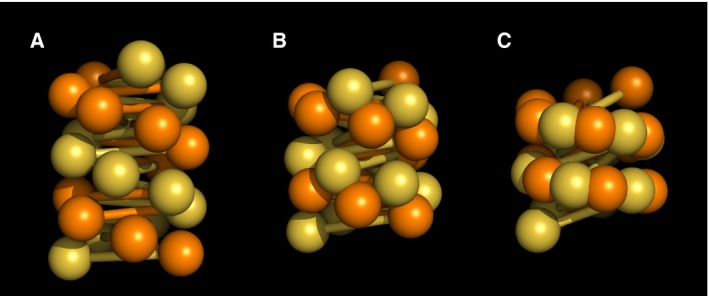
Schematic of the crossed‐linker type models. The odd and even number nucleosomes/linkers are coloured in gold and orange respectively. (A) Canonical crossed‐linker structure. (B) Intermediate structure. (C) Fully interdigitated 1‐start compact structure.

The crossed‐linker model features a precise twofold symmetry of its fibre axis with odd and even number nucleosomes resided on opposing stacks. Similarly the fibre consists two helical grooves lined with odd and even number linkers respectively (Fig. 1A).

This twofold symmetry is broken if one of the grooves narrows and concomitantly brings two nucleosome stacks into close proximity. This gives rise to a transitory structure (Fig. 1B), akin to the conversion of B‐form to A‐form DNA 23. Further, two nucleosome stacks thus positioned merge via interdigitation between nucleosomes of respective stacks. In this way, the fibre undergoes a 2‐start to 1‐start transformation (Fig. 1C).

**Figure 2:**
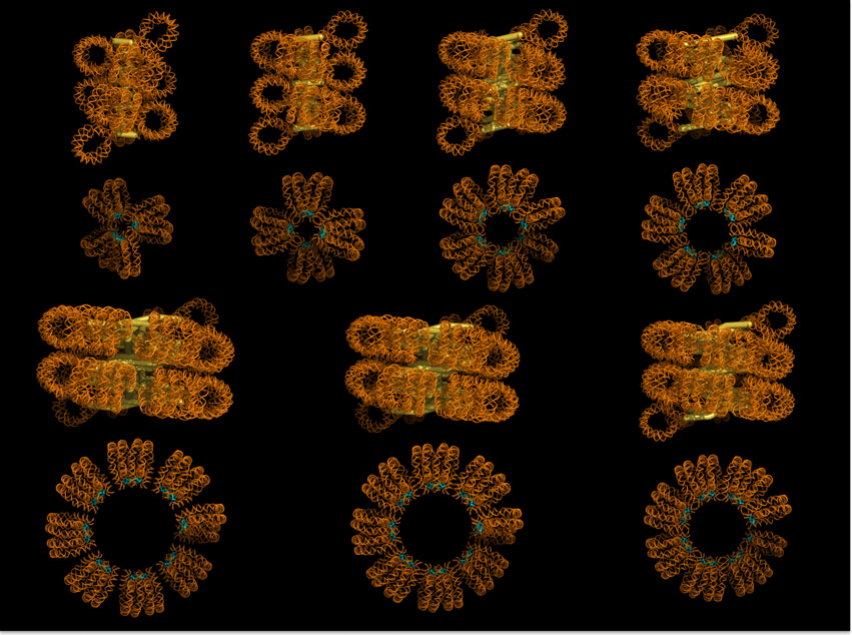
Molecular models of compact fibres corresponding to 177–237‐bp NRLs. The lower panel shows the axial view with the linker histones (H5 globular domain) highlighted in cyan. NRL, nucleosome repeat length.

The final structure envisaged here resembles the 1‐start solenoid model as originally proposed and is thus compatible with, for example, the X‐ray diffraction data. However, the crossed‐linker fibre contains largely straight linkers in contrast to tightly bent linkers in a classic solenoid. Importantly, although the two nucleosome stacks in the canonical crossed‐linker structure merge to form a single 1‐start stack in the compact structure, the two original stacks maintain their topological identity. This is because the nucleosome connectivity is still determined by the trajectory of linker DNA. Thus, although the compact fibre has the appearance and some characteristics, for example, low pitch, of a solenoid it is more accurately described as a merged 2‐start structure. Structural solutions corresponding to 177–237‐bp NRLs are illustrated in Fig. 2.

## The compact fibre is likely metastable

An important feature of the predicted structure is that nucleosomes from respective parental stacks (as in the 2‐start precursor) orient differently such that the trajectory of the nucleosome dyad axis alternates angularly along the newly formed stack (Fig. 1C), therefore also alternating the mode of interfacial interactions between adjacent octamers. In other words, because of the molecular twofold symmetry of the core particle each octamer presents reverse surface contours relative to the two adjacent neighbours. This configuration brings two opposing H2A–H2B dimers of adjacent nucleosomes closer at one interface and further apart at the other, while the reverse is true for the distance between the H4 tail and the acidic patch on the adjacent nucleosome 24. Put simply, two H2A–H2B dimers meet at one interface, while the H4 tail contacts the acidic patch at the other. In less packed stacks where optimal stacking cannot be maintained at both interfaces, near neighbours are likely formed at the better. In this situation di‐nucleosomes are apparent (Fig. 2).

Importantly alternation of dyad axes along the nucleosome stack suggests that the pseudo‐twofold symmetric interaction observed in crystal and cryo‐EM structures 5, 7 between the H2A–H2B dimers is in place (Fig. S1). Nonetheless an acute interfacial angle between adjacent nucleosomes, as is the case in fibres of smaller diameters, could in principle hinder the described interaction. The simplest solution would be for the H2A–H2B dimer to shift out of the nucleosome, as was noted in Schalch *et al*. 5; a similar mechanism was proposed elsewhere by Mozziconacci and Victor 25.

**Figure 3:**
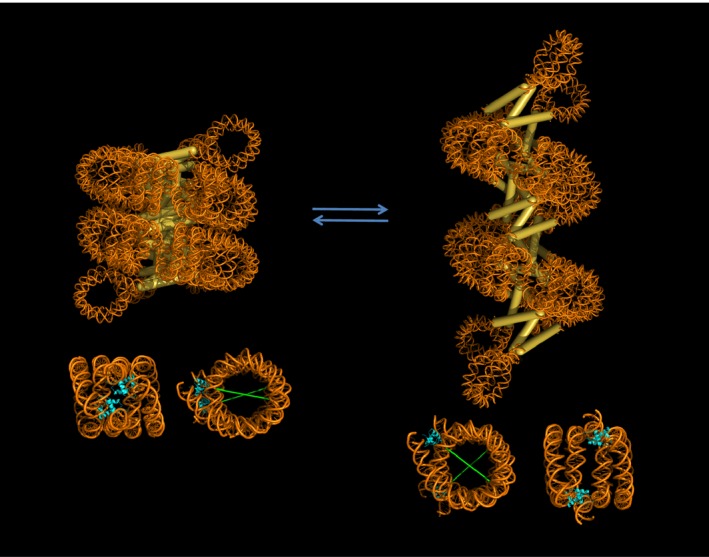
Molecular model of the 197‐bp NRL fibre in two different compaction states. Stacked nucleosomes where the opposing H2A–H2B dimers interact (see main text) are highlighted in the lower panel. At this interface respective linker histones (in cyan) form a twofold symmetry co‐axial of the interacting H2A–H2B dimers. The angle between dyad axes (in green) widens as the structure elongates. NRL, nucleosome repeat length.

The interlocking between two nucleosomal stacks means the fibre can no longer uncoil easily. In other words, there would be an activation energy barrier to uncoiling. The structure, however, allows longitudinal movement where the lengthening is accompanied by a larger separation between helix gyres (Fig. 3). This motion exaggerates the angular difference between dyad axes of adjacent nucleosomes. In this situation the pseudo‐twofold symmetry between two interacting H2A–H2B dimers may be displaced, whereas the more flexible interaction between the H4 tail and the H2A–H2B acidic patch is likely to remain. The fibre could therefore modulate the degree of compaction by assuming at different states.

The DNA topology of the proposed compact fibre structure is simply an extension of that of less compact fibres. In other words, it is a negative supercoil with multiple apices, each of which corresponds to a nucleosome. In our model the compact 1‐start form is associated with increased fibre coiling. We calculate that the constraint in the compact form of a fibre with a 197‐bp NRL corresponds to a decrease in linking number of ~ 1.7/nucleosome, a value that is quantitatively consistent with the limit compaction observed on the mechanical coiling of a fibre 26 and the negative superhelicity constrained by a chromatosome 21 (Supplementary material). The superhelical constraint from the coiling of the fibre is proportional to (1−sin γ) where γ is the angle between the linker DNA and the normal to the fibre axis. As the fibre compacts, γ decreases, and hence the fibre becomes more supercoiled. Since the compact structure results from increased coiling of the canonical crossed‐linker structure, it is a higher energy form and again likely to be metastable relative to more relaxed structures.

## Physical attributes of the proposed structure

In the most compact forms predicted of the merged 2‐start structures (177–237‐bp NRLs) the tilt of the nucleosomal disc relative to the fibre axis range between 70.3° and 75.7° (angle between the fibre axis and the normal to the nucleosomal disc). At this compaction all structures have a ~ 110 Å separation between helix gyres and a low helical pitch (5.3–11.4°). These measurements are wholly consistent with values determined from photochemical dichroism studies 10 and the X‐ray diffraction patterns of partially oriented chromatin samples 11, 12.

As the structure forms, the register between two nucleosomal stacks when interdigitation takes place varies depending on fibre diameter. The change in the register is incremental as the point of insertion among nucleosomes along the stack is incremental, for instance either between *i* and *i* + 2 or *i* + 2 and *i* + 4 and so on. Crucially, for fibres of uniform linker length this phenomenon dictates that the number of nucleosomes per turn varies by an increment of ~ 4 at each step change in the register (Fig. S3). We observed two step changes in the register as the NRL increases from 177 to 237 bp. Over this range the model algorithm gives rise to three structural classes corresponding to 177–187, 197–207 and 217–237‐bp NRLs with respectively on average 7.1, 10.9 and 15.4 nucleosomes/11 nm. These values compare with experimental determinations of ~ 6, ~ 11 and ~ 15 nucleosomes/11 nm for the packing density over the same ranges of NRL 13, 27 (Fig. S4). Since the closest possible approach of successive coils of a helical nucleosome stack is 11 nm the maximum attainable packing density/11 nm corresponds to the number of nucleosomes/helical turn. In principle in this situation the fibre diameter depends on the number of nucleosomes/turn and consequently should exhibit a similar step‐wise variation with linker length. In our calculations, although most values are close to the experimentally observed values, the step‐wise nature is less apparent, but we note that the idealised straight linker trajectory used for NRLs < 217 bp and exit‐entry angles could result in variations in diameter although not in packing density.

In a crossed‐linker model fibre compaction implies an increasingly shorter distance between the helical axes of neighbouring linker DNAs. Notably in fibres with longer linker lengths our model predicts that the closest approaches of adjacent linker DNAs in the fibre column are on average less than 2 nm (Table 1). Such duplex interpenetration has previously been observed in DNA crossovers 19 and, indeed, has been proposed as a mechanism promoting chromatin fibre compaction 18. Divalent cations facilitate right‐handed crossover formation 28, and also, in contrast to monovalent cations, promote the formation of more compact chromatin fibres 29, 30. We suggest that these close approaches of linker DNA could perhaps contribute to a relative metastability of the more compact fibres.

An important feature of the proposed structure is that although several nucleosomes at the extremity of the array do not participate in interdigitation, they could, however, interdigitate precisely with like ends to form end to end association, reminiscent of the multimeric forms of reconstituted fibres observed by Robinson *et al*. 13.

## Relation to previous work

Current models for the 30‐nm chromatin fibre assume, either implicitly or explicitly, a singular mode of interactions between adjacent nucleosomes. This is a major constraint on the structure of a molecule that has only one symmetry axis. The basic geometry of the fibre requires that the nucleosome dyads point towards the fibre core such that the linker DNA reside at the interior of the fibre. However, the assumption of uniform interactions between adjacent nucleosomes, such as between the two H2A–H2B dimers seen in the crystal structure 5, results in the dyad axes pointing radially along the nucleosomal stack because each octamer must rotate in the same sense relative to its preceding neighbour. This constraint is typical of the many models that have been proposed for the structure of the compact chromatin fibre 5, 13, 14, 15, 17. Nevertheless, Schalch *et al*. 5 showed that by increasing the negative superhelicity of the fibre the two stacks of a canonical crossed‐linker structure would approach each other and the diameter of the fibre would increase slightly. In the Schalch *et al*. model further compaction was precluded by the maintenance of dyad orientation.

The rationale for the solenoid model was based in part on the correct recognition that a solenoidal structure should have a low pitch angle 1. Indeed analysis of Necturus and chicken chromatin revealed pitch angles of 32° and 34°, respectively, for 2‐start chromatin fibres 2, 9. Similarly the cryo‐EM structure of Song *et al*. 7 has a pitch angle of ~ 40°. In contrast, the pitch angle of the merged 2‐start structure is on average ~ 8°. However, the X‐ray diffraction data of Widom and Klug 12 supporting a low‐pitch structure was obtained using hexamminecobalt as one of the condensing cations. In general such multivalent cations are more effective condensing agents than the sodium ion used in the initial studies of chromatin condensation [30, 31, 32, 33; see also 34] and would be expected to reduce the pitch angle of the chromatin fibre by favouring the closer approach of the two nucleosome stacks.

**Figure 4:**
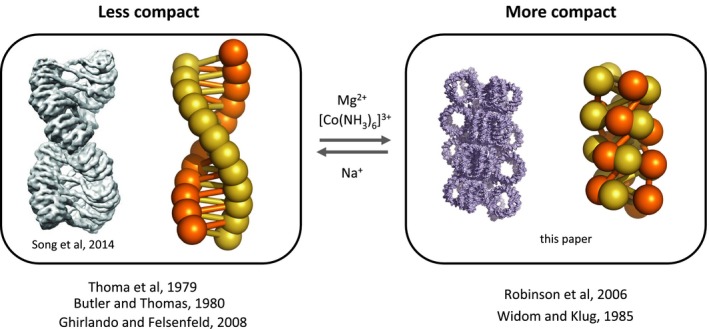
Schematic showing relation of structure to ionic environment and to previous work.

The model predicts that the negative superhelicity constrained by the fibre should increase with compaction, as experimentally observed *in vitro* 26 and suggested by the release of unconstrained negative superhelicity associated with chromatin decompaction *in vivo* 35. This result is at variance with the observation that linker histones do not increase the negative superhelicity constrained by a nucleosome array 36. However, in these latter experiments only Na^+^ ions were present and consequently full compaction would not be attained. Similarly the absence of divalent cations could potentially restrict the fibre packing density to ~ 6 nucleosomes/11 nm, as frequently observed (e.g. 37; Fig. 4).

## Concluding remarks

We have argued that a compact 1‐start crossed‐linker chromatin fibre is consistent with the currently available experimental data. In particular it is a property of the model that the interdigitation of two nucleosome stacks predicts that the number of nucleosomes/turn, and by extension the packing density of the fibre, should increase in increments of ~ 4 nucleosomes/turn. This is consistent with the data of Robinson *et al*. 13 and is not predicted by other models. Further the model is applicable to quantized NRLs in the range of 177 bp up to 237 bp. We note that the model does not predict the most energetically stable form of the fibre and since it is necessarily in equilibrium with more relaxed forms the position of that equilibrium is likely linker‐length dependent.

The key to the compact structure is that the fundamental structural unit is not a nucleosome but a dinucleosome. If one constructs the nucleosomal stack using a dinucleosome as the structural unit the problem is simply resolved. In the proposed structure interactions between adjacent H2A–H2B dimers stabilise the dinucleosome while the H4 tail provides the linkage between dinucleosomes.

The model we have presented is one of a class of models in which a 2‐start structure forms a 1‐start structure by interdigitation. During the construction of physical models it has also become clear to us that the structure needs only sporadic interdigitations to hold, due to its interlocking mechanism. In fact, as few as three stabilising interactions (either the intra‐ or interunit linkage) per turn suffice to maintain the structure. Further, interdigitation can occur with arrays of mixed NRLs. These findings reveal that uniformity of linker length is not a necessity for this structure.

## Author contributions

CW: co‐conceived hypothesis, structural modelling, cowrote paper. JM: structural modelling, edited paper. AT: co‐conceived hypothesis, DNA topology modelling, cowrote paper.

## Supporting information


**Data S1.** Supplementary 1, Supplementary 2 (complete legends of Figs S1–S4), Supplementary 3 (complete caption of Table S1 and Supplementary references, Supplementary S4 (video file), and Supplementary S5.
**Fig. S1.** The relative orientations of the adjacent nucleosomes facilitate the pseudo‐twofold symmetric interaction between the opposing H2A–H2B dimers.
**Fig. S2.** Chromatosome models in the merged 2‐start compact fibres.
**Fig. S3.** Interdigitation patterns between nucleosome stacks of the 2‐start crossed‐linker structure.
**Fig. S4.** Correspondence between calculated and experimentally determined packing densities (A) and diameters (B) of compact fibres.

**Table S1.** Comparison of calculated parameters for a compact 197‐bp NRL fibre with experimental values obtained with fibres with NRLs in the range of ~ 190–210 bp.

**Video S1.** Compaction and decompaction of a 197‐bp NRL fibre.
