# Supplementary material for: A metastable structure for the compact 30‐nm chromatin fibre
Source: FEBS Lett. 2016 Mar 30;590(7):935–42. doi: 10.1002/1873-3468.12128 (PMC4863496; doi:10.1002/1873-3468.12128)
Supplement: Supplementary file 1 — Data S1. Supplementary 1, Supplementary 2 (complete legends of Figs S1–S4), Supplementary 3 (complete caption of Table S1 and Supplementary references, Supplementary S4 (video file), and Supplementary S5. Fig. S1. The relative orientations of the adjacent nucleosomes facilitate the pseudo‐twofold symmetric interaction between the opposing H2A–H2B dimers. Fig. S2. Chromatosome models in the merged 2‐start compact fibres. Fig. S3. Interdigitation patterns between nucleosome stacks of the 2‐start crossed‐linker structure. Fig. S4. Correspondence between calculated and experimentally determined packing densities (A) and diameters (B) of compact fibres. [file FEB2-590-935-s001.pdf]

Supplementary material

Supplementary 1:

Topology of compact fibre

Calculated value of superhelical constraint/nucleosome in compact fibre.

Derivation from model:

The calculation treats the trajectory of DNA in a 30 nm fibre as an uncapped plectoneme with multiple apices. Each apex corresponds to a core nucleosome while the linker DNA and the associated crossovers (interwindings) contribute to the compaction of the fibre.

Total constraint must be of the form:
$C_{total} = C_{apices} + C_{interwindings}$

Increase in superhelical density is equivalent to an increase in torque or twist. This means that because in a plectoneme
$Tw = -n(1-\sin\gamma)$ where γ is the average pitch angle of the linker DNA and n the number of turns.
Therefore $C_{interwindings}$ must be proportional to $(1-\sin\gamma)$.
However because the crossed-linker structure is not capped (i.e. is open) the number of turns is not n but n/2 relative to the apparent number (Cozzarelli et al, 1990).

From this:
Constraint/crossover ≈ constraint/nucleosome because there is 1 crossover/nucleosome and crossovers determine extent of rh coiling
i.e. $C_{nucleosome} = C_{apex} - (1-\sin\gamma)/2$

Putting in real numbers:
approximating $\gamma = 0°$ at maximum compaction
$\Delta L_{nucleosome} = -1.1 - 0.5 = -1.6$ or, taking experimental limits,
$-1.2 – 0.5 = -1.7$

Experimental values for C_{apex} from Zivanovic et al (1986),
$\Delta L_{nucleosome}$ compares with -1.6 to -1.7 from Zivanovic et al (1990).

Note that as the fibre compacts γ decreases and consequently the constrained superhelicity increases (see also Norouzi and Zhurkin, 2015).

In vitro - data from Recouvreux et al. (2011):
Estimate of constraint by applying negative torque to 38 x 200 bp nucleosome array with 601 repeats.
For a single array of 38 nucleosomes maximum compaction at ~ 66 lh turns.
Therefore additional superhelicity due to coiling associated with compaction
$= -(66 – 38)/38 = -0.71$/nucleosome
Total constraint / nucleosome: $\Delta L = -1.71$

Average superhelical density / nucleosome $= -1.71\ (10.5/200)$
$= -0.089$

Supplementary 2:

Figure S1 The relative orientations of the adjacent nucleosomes facilitate the pseudo-two-fold symmetric interaction between the opposing H2A-H2B dimers. Left panel: superimposition of stacked nucleosomes from models of 177 – 237 bp NRLs. The structures were built using core particles alone (PDB ID: 3LZ0; Vasudevan et al, 2010) for a direct comparison with the crystal structure (Schalch et al, 2005). Right panel: the model of the two stacked nucleosomes built in accordance to the tetranucleosome crystal structure (Schalch et al, 2005).

Figure S2 Chromatosome models in the merged 2-start compact fibres. Upper panel: superimposition of chromatosome models with varying entry/exit points corresponding to 177-237 bp NRL fibres. Lower panel: crystal structure of the chromatosome (Zhou et al, 2015).

Figure S3 Interdigitation patterns between nucleosome stacks of the 2-start crossed-linker structure. For uniform linkers in fibres of perfect symmetry, the number of nucleosomes per turn can only feasibly change by a 4-nucleosome step, unless the linker length is allowed to vary as shown in the case of 10 nucleosomes/turn. This implies that both packing density and fibre diameter should exhibit similar step-change patterns, providing same separation between successive gyres and correlation of fibre circumference with the number of nucleosomes per turn.

Figure S4 Correspondence between calculated and experimentally determined packing densities (Figure S4a) and diameters (Figure S4b) of compact fibres. Experimental data (in black) taken from Robinson et al. (2006). Calculated data (in red) from this paper. Note that there is good correspondence between the calculated and experimental data except for the 177 bp NRL data for both packing density and fibre diameter.

Supplementary 3:

Table S1 Comparison of calculated parameters for a compact 197 bp NRL fibre with experimental values obtained with fibres with NRLs in the range of ~190-210 bp

Supplementary references:

Cozzarelli NR, Boles TC, White JH (1990) Primer on the topology and geometry of DNA supercoiling. In DNA topology and its biological effects (Cozzarelli NR, Wang JC, eds) pp 139-184, Cold Spring Harbor Laboratory Press, Cold Spring Harbor, New York.

Frouws TD, Patterton HG, Sewell BT (2009) Histone octamer helical tubes suggest that an internucleosomal four-helix bundle stabilizes the chromatin fiber. Biophys J 96: 3363-3371.

Ghirlando R, Felsenfeld G (2008) Hydrodynamic studies on defined heterochromatin fragments support a 30 nm fiber having 6 nucleosomes per turn. J Mol Biol 376: 1417-1425.

Luger K, Mäder AW, Richmond RK, Sargent DF, Richmond TJ (1997) Crystal structure of the nucleosome core particle at 2.8 A resolution. Nature 389: 251-260.

McGhee JD, Rau DC, Charney E, Felsenfeld G (1980) Orientation of the nucleosome within the higher order structure of chromatin. Cell 22: 87-96.

Norouzi D, Zhurkin B (2015) Topological polymorphism of the two-start chromatin fibre. Biophys J 108: 2591-2600

Recouvreux P, Lavelle C, Barbi M, Condé e Silva N, Le Cam, E, Victor JM, Viovy J L, (2011) Linker histones incorporation maintains chromatin fiber plasticity. Biophys J 100: 2726–2735.

Robinson PJJ, Fairall L, Huynh VAT, Rhodes D (2006) EM measurements define the dimensions of the "30-nm" chromatin fibre: Evidence for a compact, interdigitated structure. Proc Nat Acad Sci USA 103: 6506-6511.

Schalch T, Duda S, Sargent DF, Richmond TJ (2005) X-ray structure of a tetranucleosome and its implications for the chromatin fibre. Nature 436: 138-141.

Sen D, Mitra S, Crothers DM (1986) Higher order structure of chromatin: Evidence from photochemically detected linear dichroism. Biochemistry 25: 3441-3447.

Song F, Chen P, Sun D, Wang M, Dong L, Liang D, Xu RM, Zhu P, Li G (2014) Cryo-EM study of the chromatin fibre reveals a double helix twisted by tetranucleosomal units. Science 344: 376-380.

Vasudevan D, Chua EY, Davey CA (2010) Crystal structures of nucleosome core particles containing the '601' strong positioning sequence. J Mol Biol 403: 1-10.

Widom J (1986) Physicochemical studies of the folding of the 100 Å nucleosome filament into the 300 Å filament. Cation dependence. J Mol Biol 190: 411-424.

Zhou BR, Jiang J, Feng H, Ghirlando R, Xiao TS, Bai Y (2015) Structural mechanisms of nucleosome recognition by linker histones. Mol Cell 59: 628-638.

Zivanovic Y, Goulet I, Revet B, Le Bret M, Prunell A. (1988) Chromatin reconstitution on small DNA rings. I. J Mol Biol 200: 267-920.

Zivanovic Y, Duband-Goulet I, Schultz P, Stofer E, Oudet P, Prunell A (1990) Chromatin reconstitution on small DNA rings. III. Histone H5 dependence of DNA supercoiling in the nucleosome. J Mol Biol 214: 479-495.

Supplementary 4

File 197.mp4

File illustrates compaction and decompaction of a 197 bp NRL fibre.

Supplementary 5

Pymol PSE files for 197 bp NRL fibre (197 fibre.pse) and for extended 197 bp fibre (197 extended.pse).

Figure S1

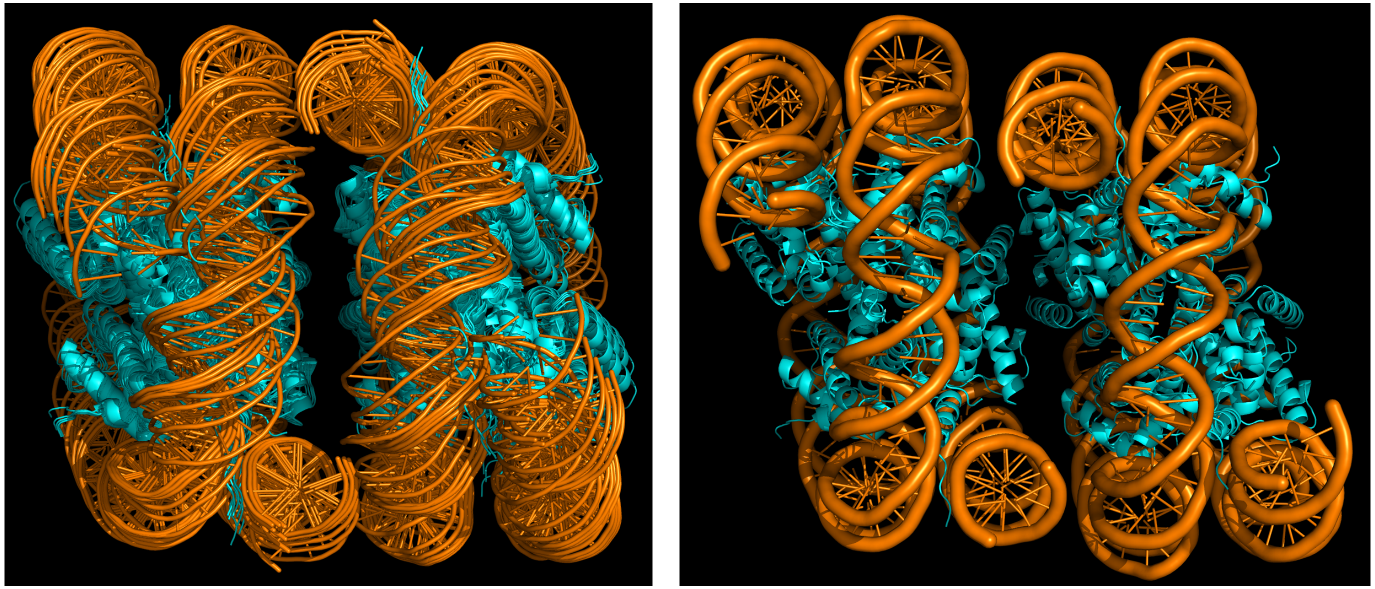

Figure S2

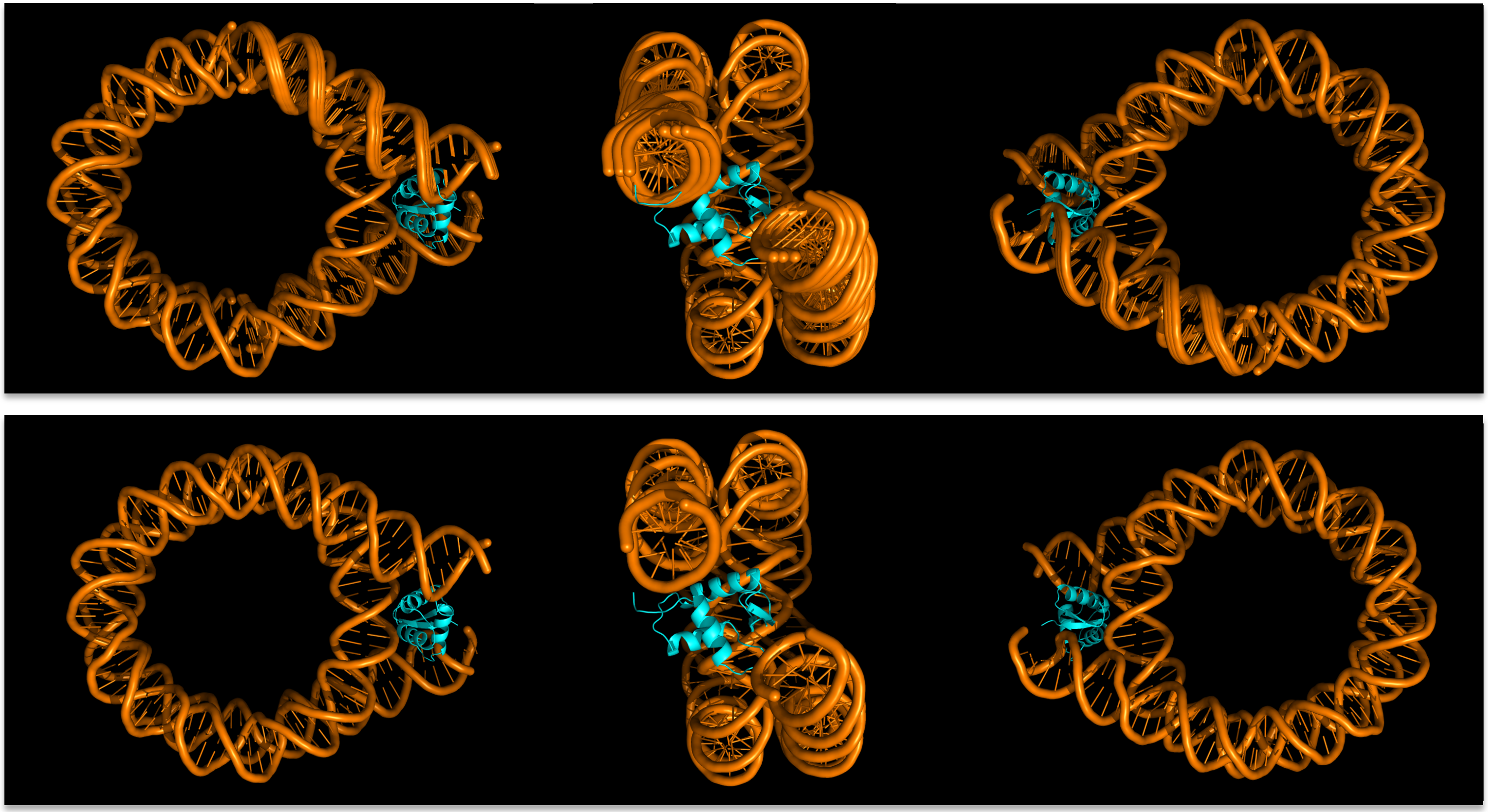

Figure S3

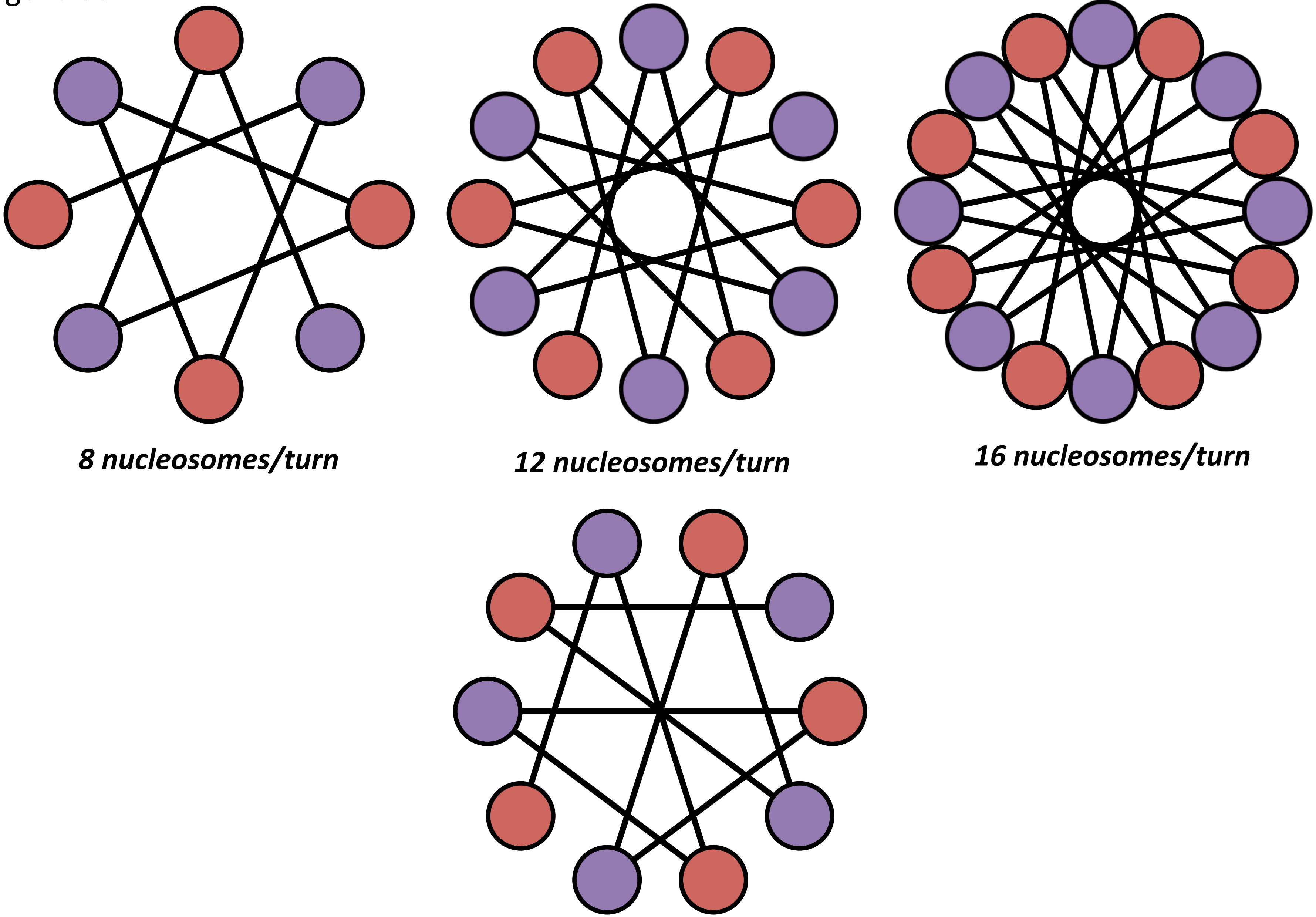

8 nucleosomes/turn

12 nucleosomes/turn

16 nucleosomes/turn

10 nucleosomes/turn

Figure S4a

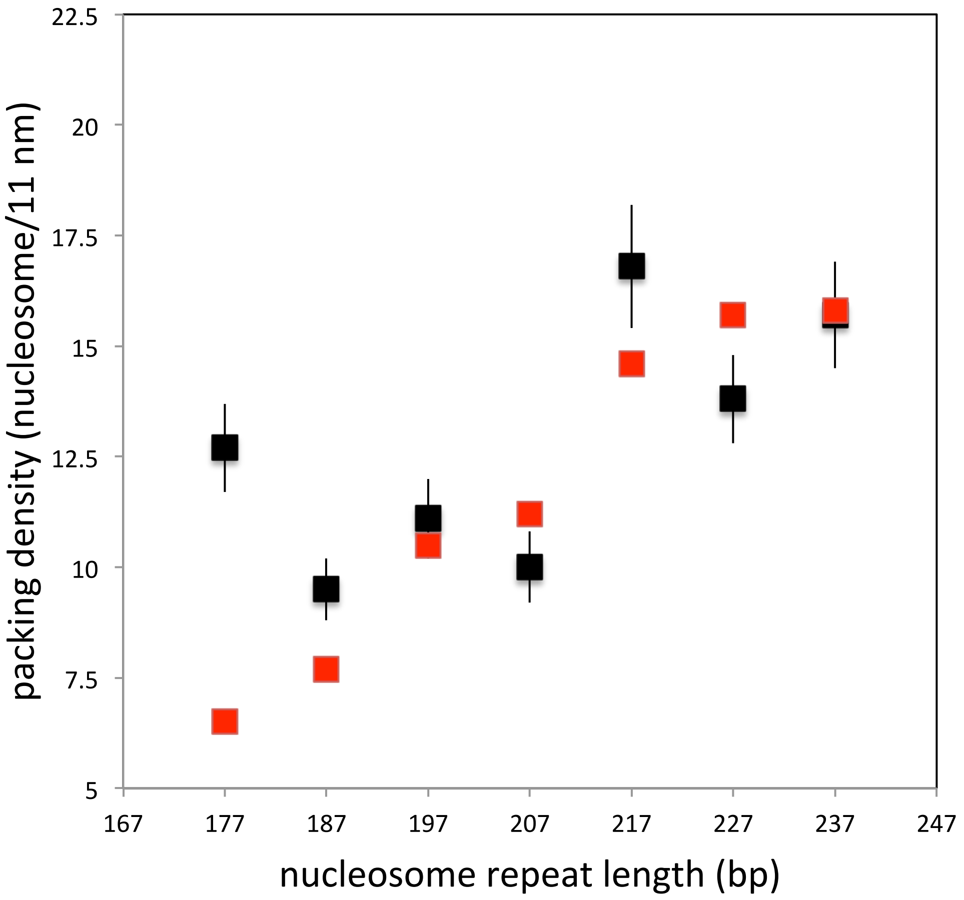

22.5
20
17.5
15
12.5
10
7.5
5
packing density (nucleosome/11 nm)
167
177
187
197
207
217
227
237
247
nucleosome repeat length (bp)

Figure S4b

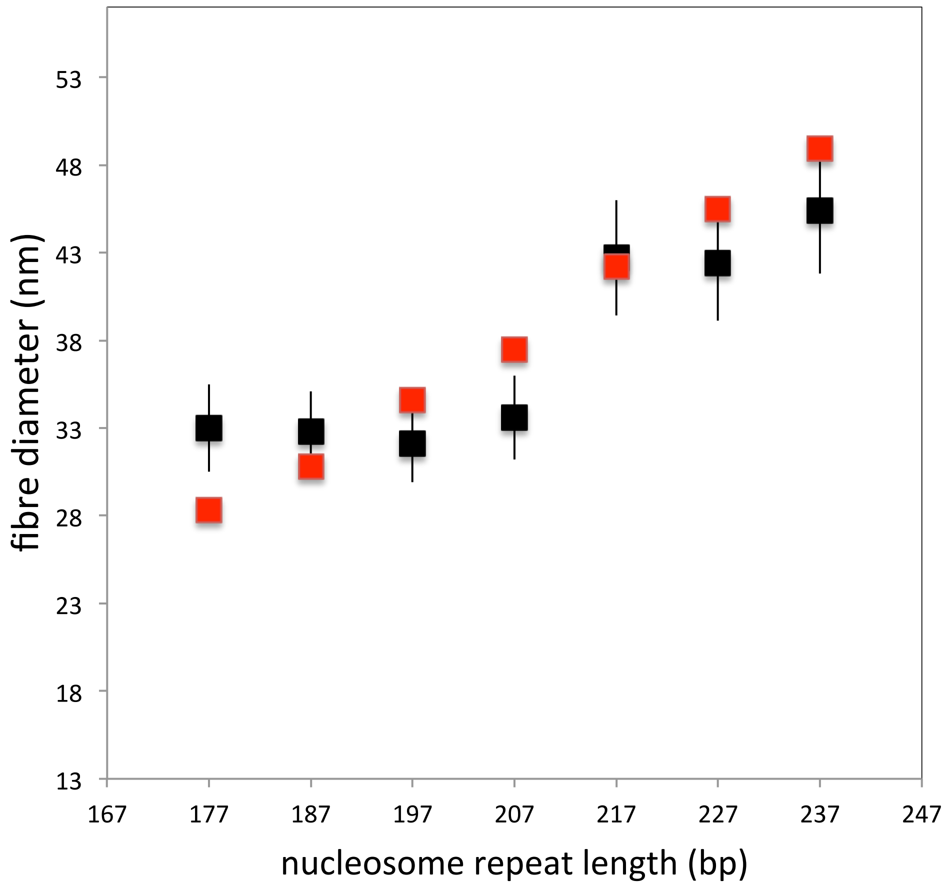

fibre diameter (nm)
53
48
43
38
33
28
23
18
13
167
177
187
197
207
217
227
237
247
nucleosome repeat length (bp)
