# Supplementary material for: A metastable structure for the compact 30‐nm chromatin fibre
Source: FEBS Lett. 2016 Mar 30;590(7):935–42. doi: 10.1002/1873-3468.12128 (PMC4863496; doi:10.1002/1873-3468.12128)
Supplement: Supplementary file 2 — Table S1. Comparison of calculated parameters for a compact 197‐bp NRL fibre with experimental values obtained with fibres with NRLs in the range of ~ 190–210 bp. [file FEB2-590-935-s002.docx]

**Table S1**

Relaxed nd Compact

(archetypal 30 nm fibre) model (197 bp NRL) experimental

Fibre dimensions:

Diameter 30-32 nm 34 nm 32 nm (1)

Packing density ~6 nuc/11 nm (2,3) 12 nucs/11 nm 11 nucs/11 nm (1)

Nucleosome inclination 20-33° (4) 30-40° (5) 28°

Superhelical constraint (ΔL/nucleosome) -1.6 to -1.7 -1.6 (6)

Pitch ‘low’ (7) 8.3°

Stepwise variation with linker length

Packing density Yes Yes (1)

Diameter See discussion Yes (1)

Internucleosomal interactions H4-H2A (8) Yes

H2A-H2A (9,10) Yes

References:

1. Robinson et al.

2. Ghirlando & Felsenfeld

3. Song et al

4. McGhee et al

5. Sen et al

6. Recouvreux et al.

7. Widom & Klug

8.Luger et al.

9. Schalch et al

10. Frouws et al
